# Use of IMU in Differential Analysis of the Reverse Punch Temporal Structure in Relation to the Achieved Maximal Hand Velocity

**DOI:** 10.3390/s21124148

**Published:** 2021-06-17

**Authors:** Stefan Marković, Anton Kos, Vesna Vuković, Milivoj Dopsaj, Nenad Koropanovski, Anton Umek

**Affiliations:** 1 Faculty of Electrical Engineering, University of Ljubljana, 1000 Ljubljana, Slovenia; anton.kos@fe.uni-lj.si (A.K.); anton.umek@fe.uni-lj.si (A.U.); 2 Faculty of Sport and Physical Education, University of Belgrade, 11000 Belgrade, Serbia; vbvukovic@gmail.com (V.V.); milivoj.dopsaj@gmail.com (M.D.); 3 Institute of Sport, Tourism and Service, South Ural State University, 454080 Chelyabinsk, Russia; 4 Department of Criminalistics, University of Criminal Investigation and Police Studies, 11000 Belgrade, Serbia; korpan82@gmail.com

**Keywords:** IMU, karate, punch velocity, gyaku zuki, event timeline, accelerometer, gyroscope, sensor fusion

## Abstract

To achieve good performance, athletes need to synchronize a series of movements in an optimal manner. One of the indicators used to monitor this is the order of occurrence of relevant events in the movement timeline. However, monitoring of this characteristic of rapid movement is practically limited to the laboratory settings, in which motion tracking systems can be used to acquire relevant data. Our motivation is to implement a simple-to-use and robust IMU-based solution suitable for everyday praxis. In this way, repetitive execution of technique can be constantly monitored. This provides augmented feedback to coaches and athletes and is relevant in the context of prevention of stabilization of errors, as well as monitoring for the effects of fatigue. In this research, acceleration and rotational speed signal acquired from a pair of IMUs (Inertial Measurement Unit) is used for detection of the time of occurrence of events. The research included 165 individual strikes performed by 14 elite and national-level karate competitors. All strikes were classified as slow, average, or fast based on the achieved maximal velocity of the hand. A Kruskal–Wallis test revealed significant general differences in the order of occurrence of hand acceleration start, maximal hand velocity, maximal body velocity, maximal hand acceleration, maximal body acceleration, and vertical movement onset between the groups. Partial differences were determined using a Mann–Whitney test. This paper determines the differences in the temporal structure of the reverse punch in relation to the achieved maximal velocity of the hand as a performance indicator. Detecting the time of occurrence of events using IMUs is a new method for measuring motion synchronization that provides a new insight into the coordination of articulated human movements. Such application of IMU can provide additional information about the studied structure of rapid discrete movements in various sporting activities that are otherwise imperceptible to human senses.

## 1. Introduction

Karate is a high-intensity combat sport that imposes high psychophysical and physiological demands on the athlete [1,2]. It involves repeated explosive execution of technically demanding strikes [3], which are used to score points and develop tactical advantage [4]. The importance of hand strikes in karate is underlined by the fact that they account for more than 80% of all points scored in competitive matches, with the most commonly used strike being the reverse punch [5,6]. In the dynamically changing conditions of a competitive match, the reverse punch is a versatile tool that can be employed efficiently as a direct attack, interception, or counterattack. 

The reverse punch is a fundamental technique taught to all karate practitioners from the very beginning of their training. It is executed from a guard position, with the hand opposite to the lead leg [7]. The force of the strike is mainly contributed by the drive off the ground by the legs, rotation of the trunk, and action of the arm muscles [4,8]. In relation to the inter-joint coordination, the motion is characterized by a consecutive proximal-to-distal sequencing [9,10], which enables the hand to be imparted with the energy of the preceding motion. This is a pattern found in different striking or throwing-like movements [11] and is considered essential for generating high velocities at the endpoint of the kinetic chain, in this case, the fist. However, such complex motor action requires sequential control of the series of movements [12] with optimal intra and inter-muscular coordination [13]. The temporal structure of the punch represents the invariant aspect of the generalized motor program [14] governing the execution of the strike. It is affected by the motor learning strategies that result in alterations in the internal processes that determine an individual capacity to produce a motor action after practice [15]. Thus, permanent and periodical monitoring of the athletes’ technique can be considered a precondition for unhampered progression toward efficient execution of the technique in competitive settings [16,17].

The previous studies have addressed the kinematics of the striking motions, as well as the underlying neuro-mechanics, using primarily optical 3D motion capture systems and electromyography. This requires costly specialized equipment along with trained technicians to operate it, making it less accessible to the majority of the coaches and athletes. An additional constraint for widespread use of motion capture systems (QTM, Vicon etc.) in sport praxis is the time required to setup the system for a single athlete, let alone for a group of athletes. Consequently, coaches remain unable to objectively quantify the changes in the athletes’ technique and subjective evaluation remains the predominant ‘method’ used in praxis [18,19]. Such an approach can lead to significant conceptual errors in training introduced by a coach misjudging some relevant aspect of the athletes’ motion.

However, with the recent technological advances, micro-electromechanical sensor systems (MEMS) are becoming more widely implemented for the purposes of obtaining more sensitive and sport-specific information (compared to human observation commonly used in sport praxis) in relation to the level of achieved preparedness in athletes [20,21]. In this sense, fairly recent papers [18,22] point to the possibility of applicable use of IMUs (Inertial Measurement Unit) in combat sports, while similar solutions have been widely developed and implemented in other sport disciplines. Papers [23,24] have shown that IMUs can be used to provide information on different phases of the movement in baseball pitching and golf swing, respectively, while a paper [25] provides an exemplary overview on the use of inertial sensors for the purposes of human motion tracking. This indicates that such measurement equipment and related software solutions can be efficiently used to provide concurrent or terminal feedback to the coaches and athletes [26]. This, in turn, can lead to the objectification of training methods and improvements in athletes’ technical proficiency, thus contributing to the advancement of the competitive results. IMUs are employed in integrated systems with different complexity, where for general purpose applications large numbers of sensors are used to cover the predefined body attachment points, i.e., anatomical landmark positions, while for specific well-defined movements the number of sensors should be reduced to a considerably lower number. An important feature of specific solutions includes an easier equipment use, primarily in regard to its calibration and setup, while user experience is related to a specific software implementation of the user interface. They provide a sufficient level of precision for measurement of human movement kinematics, especially for measurement of time-related characteristics of rapid movements. However, it should be stressed that the main reason for the selection of IMUs is that they provide sufficient information on the examined movements in terms of being able to detect changes in their kinematic and temporal structure otherwise undetectable to human senses, while not compromising the regular training conditions and workflow.

This paper aims to determine the differences in the temporal structure of the reverse punch, as measured by a pair of IMU, in relation to the achieved maximal velocity of the hand. We hypothesize that the differences exist in the order of the detected events between the punches classified into different groups according to the achieved maximal velocity of the hand. The time of occurrence of the events that are describing the structure of the punch are defined as acceleration and rotational speed threshold values, peaks, and zero crossings.

The IMU sensors-based measurement system in our study is not used in a classical way for motion tracking, but for the detection of the sequence of events gathered from acquired sensor signals. Sensor inaccuracies have practically no effect on the detection of the sequence of events. Our claim is based on our previous research papers [27,28,29], which study in detail the inaccuracies of accelerometers and gyroscopes and provide guidelines for their use in various applications, including those that use human motion and its kinematic variables. The focus is on guidelines for the proper use of inertial sensors in applications that use measured and/or calculated kinematic variables in sports activities. The main findings are that the sensor noise and bias can only be reduced to a certain extent by methods such as filtering and bias removal. The results of the analysis of the influence of accelerometer and gyroscope inaccuracies on the event times confirm that the error in time measurement does not exceed one sampling time. In the proposed methodology, the main result of the timing analysis is the order of events, which means that a minor error in the timing measurement usually does not affect the final result, i.e., the detected sequence of events. For this reason, we argue that the method used for the detection of the sequence of events is not sensitive to sensor inaccuracies. More details about the influence of sensor inaccuracies on sequence of detected events can be found in Section 2.3.

The underlying concept of the study employs the simple idea of a minimalistic, easy-to-use, robust system setup that aims to provide feedback in relation to a specific key feature of the movement that can affect its outcome. Such an approach overcomes the need for a full kinematic analysis or a more complex sensor setup because it provides sufficient data on the motion synchronization problem it is intended to address. 

The major scientific contributions of the paper are: (a) a novel methodology for measuring the temporal sequence of the movement, which is based on the application of IMUs for motion sequence acquisition, implicitly considering the synchronization of movement sub-elements as detected using the time of occurrence of relevant events in the signal; (b) a more detailed explanation of sensor inaccuracies and a demonstration that they do not affect the temporal sequence acquisition based on events extracted from kinematic variables of sensor signals; (c) establishing the differences between groups in terms of the temporal structure of the movement based on the maximal hand velocity as an objective performance measure; (d) providing an initial model of the optimal temporal structure of the movement using the presented methodology.

## 2. Materials and Methods

### 2.1. The Research Sample

The sample in this research consisted of 14 elite and national level karate kumite competitors (age: 20.33 ± 2.15 years; body height: 1.85 ± 0.03 m; body mass: 81.33 ± 5.03 kg; training experience: 7.23 ± 2.36 years) who performed a total of 165 individual strikes across multiple testing sessions conducted according to the availability of the athletes in the interval of May–November 2019. Consequently, individual testing sessions correspond to different theoretical phases of the yearly training cycle of karate athletes, which added to the variability of the measured results as the performance of an individual change due to the applied training methods.

The actual testing was preceded by an individual warm-up (15 min), after which each participant executed 3 test trials separated by a 1 min pause. Each trial consisted of a single reverse punch performed from the preferred side fighting stance (Fudo dachi) with the back leg bent, hips positioned at the angle relative to the punch direction, and arms in guard position [7]. This allowed for full utilization of the entire kinetic chain during the strike [4,30]. The participants were instructed to execute the strike with maximal intensity, aiming to achieve the highest possible velocity of the hand. Prior to the testing, all subjects were informed in detail about the measurement procedures and the possible risks and benefits of this research. The study was conducted in accordance with the postulates of the Declaration of Helsinki and was approved by the Ethics Committee of the University of Belgrade Faculty of Sport and Physical Education (02 No. 484-2).

### 2.2. Measurement Method

The measurement of the movement kinematics was performed using a modified measurement system previously used in [21,31,32]. The system can support multiple wireless sensor devices connected to a laptop running the main application developed in the LabView software environment (LabView 2019, National Instruments, Austin, TX, USA). The main purpose of the application is to enable signal acquisition as well as real-time control and synchronization of the sensor devices. This is achieved by the utilization of the UDP communication protocol.

**Figure 1:**
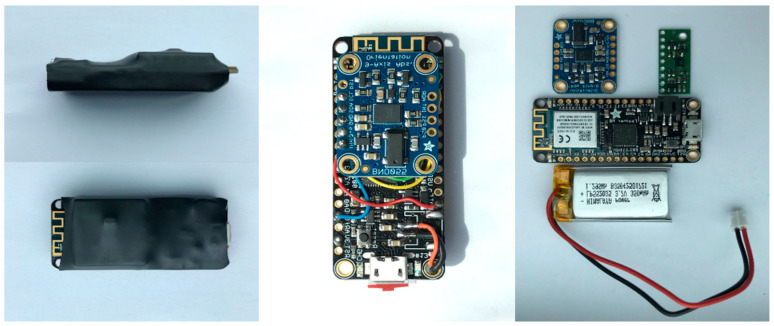
Sensor unit packed in a protective housing, fully assembled, and disassembled (from left to right).

In this research, we used two custom-made wireless sensor devices employing a 6 DOF LSM6DS33 [33] 3D accelerometer/gyroscope and a 9 DOF Bosch BNO055 [34] orientation sensor, mounted on an Adafruit Feather M0 WiFi micro-controller with a built-in communication module [35], all powered by a LiPo battery and packed in a protective housing. The LSM6DS33-based unit sampling frequency was set to 200 Hz and the BNO055-based unit had a sampling frequency of 100 Hz. The accelerometer measurement range is up to ±16 for LSM6DS33 and ±4 g_0_ BNO055, respectively. The BNO055 excludes gravitational acceleration, as its vector can be calculated and subtracted from acceleration, taking into account the orientation of the sensor derived from multiple sensor signals (accelerometer, gyroscope, magnetometer) by sensor fusion algorithm. Thus, BNO055 provides linear acceleration as defined in the manufacturer datasheet [34]. The gyroscope measurement range is up to ±2000 deg/s, for both LSM6DS33 and BNO055. The BNO055 magnetometer measurement range is ±1300 μT (x-axis, y-axis) and ±2500 μT (z-axis) [34]. Orientation is calculated at a frequency of 100 Hz. Figure 1 shows one of the sensor units used for the purposes of this research.

**Figure 2:**
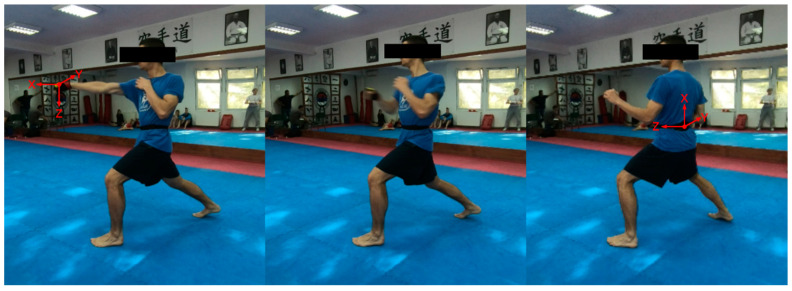
The movement start, transition, and end position with the positioning of the IMU and orientation of the sensor axes.

The sensor devices were placed on the athletes’ lower back and hand, more precisely on the area corresponding to the lumbar vertebrae IV and V (BNO055) and the dorsal side of the athletes’ hand between metacarpal bones II to IV (LSM6DS33). The positioning of the devices was chosen in order to provide relevant data regarding the acceleration and rotational speed of the center of gravity of the body and the fist of the striking hand, respectively. The sensor device was fixated on the back of the athlete using an elastic strap [36], while the sensor on the hand was embedded into the elastic glove tightly fitting the hand. The BNO055 unit allows for orientation calculation with a drawback of lower sampling frequency. Thus it was considered suitable for monitoring the movement of the athlete’s body. As the hand movement is considerably more intensive we used LSM6DS33 on the hand as it allows for higher sampling frequency. Figure 2 shows the performed movement starting (right), transition (middle), and final (left) position, with the positioning of the IMU and orientation of the sensor axes.

Sensor calibration before measurements included bias compensation. Bias measurement was performed with sensor devices in a standstill position in a controlled vertical plane with the bias measurement averaging an interval of 10 s. Although the two sensor devices are similar, they have a different IMU sensor chip installed and a different sampling frequency, as mentioned above. Signal samples from both sensor devices are received in separate LabView application program loops. The main LabVIEW program loop with a controlled timing cycle of 5 ms reads the currently available data from both sensor devices. As the UDP protocol does not prevent the data loss imposed by packet collisions on a high-loaded ISM band, possible lost data samples are replaced with their previous values. The channel quality monitoring is used to confirm the validity of measurement results. 

In the post-processing phase, the LSM6DS33 signal was filtered using a 5th order Butterworth low-pass filter with a 40 Hz cutoff frequency while the BNO055 signal was used as acquired [31]. A custom MathCad 7 script employing peak and threshold detection was developed based on the pilot results in order to extract the time of occurrence of relevant movement events identified from acceleration and rotational speed signals. Threshold values for acceleration and rotation speed are determined experimentally from the obtained measurement results. The threshold values are in line with 3–5% of the maximal value that is regularly used in the biomechanical analyses of human motion. This research considered the timeline of events preceding the point where the strike was delivered to the target, which was identified as the peak in the absolute value of hand acceleration [21,31,32]. Due to the specific movement pattern of the reverse punch performed from the front stance previously described in [7], all events were acquired from the primary movement axis of the hand, i.e., the *X* axis, as well as the primary and vertical movement axes of the body, i.e., *Z* and *X* axes, respectively.

### 2.3. Analysis of Sensor Inaccuracies

We must emphasize that our measurement system with IMU sensors is not used in a classical way for motion tracking. Sensors are not used for accurate analysis of movements in space, but to detect the timing of selected events in specific movements during the execution of the gyaku-zuki karate punch. The events are defined based on the selected characteristics of the measured acceleration and angular velocity signals as presented in Table 1. Events are defined by detecting different signal characteristics: extrema and threshold crossings. The time measurement resolution is primarily limited by the signal processing sampling time, but can also be affected by sensor bias and sensor noise.

While threshold transition times are sensitive to sensor noise and bias, extrema are only sensitive to sensor noise. The analysis of the effect of sensor noise and bias on event time measurement in Table 1 can be limited to errors for one sample time. A timing error occurs when the amplitude disturbance is greater than the change in the value of the signal between adjacent samples observed at the time of event. To obtain an accurate answer, we measured the amplitude disturbances of the sensors (bias and noise) and analyzed the sample-to-sample differences of the signal near the points of all measured events defined in Table 1.

Most of the sensor bias can be removed, but a smaller part remains due to bias drift after compensation. In our study, the sensors were calibrated before attachment to the athlete’s body, and the measured drifts of the accelerometer and gyroscope bias did not exceed 3 mg_0_ and 0.1 dps, respectively. The noise of the accelerometer and gyroscope, unlike their biases, actually limits their accuracy; typical values of the noise constants, ARW (Angle Random Walk) and VRW (Velocity Random Walk), are provided by the manufacturer of the sensor. Our sensor noise data are based on measurements of sensor signals in the state of complete physical quiescence of the sensors. All sensor signals are first filtered with a low-pass filter (Butterworth, *N* = 5, *f_cof_* = 40 Hz). The shift of the detected events for one sample is influenced by the difference of adjacent noise samples. The noise measurements show that the maximal difference of adjacent noise samples of tested accelerometers does not exceed 5 mg_0_, and the maximal difference in adjacent noise samples of tested gyroscopes is less than 0.3 dps.

We calculated the difference of adjacent signal samples near all characteristic points associated with the events in Table 1. The sample-to-sample differences of the measured signals in most of the characteristic points are more than ten times larger than the sample-to-sample differences of sensors noise. The results of the analysis of the influence of accelerometer and gyroscope errors on the event times confirm that the error in time measurement practically does not exceed one sampling time. The exception is the error in measuring the time of the motion start event (V_A_D), which in any case occurs as the first event in the chain. 

In the proposed methodology, the main result of the timing analysis is the order of the sequence of events, which means that a minor error in the timing measurement usually does not affect the final result. Therefore, a slight influence of the sensor error on the intermediate result of the measured event times has an insignificant effect on the correct detection of the sequence of events. For this reason, we argue that the method used for the detection of the sequence of events is not sensitive to sensor inaccuracies.

### 2.4. Events

The events used in this research are extracted from signals acquired from two sensor devices placed on the hand (HAND) and back (BACK) of the athlete. Temporal events are ranked in the movement timeline. Due to its relationship with kinetic energy of the strike [7], the maximal hand velocity was used as a performance indicator and a basis for group division. All relevant information regarding the temporal events is presented in Table 1. In the used system of abbreviations, (X_Y_Z) X refers to the hand (H), body (B), or vertical (V); the Y character/set of characters refers to the origin, and the Z character refers to the detected instance.

**Figure 3:**
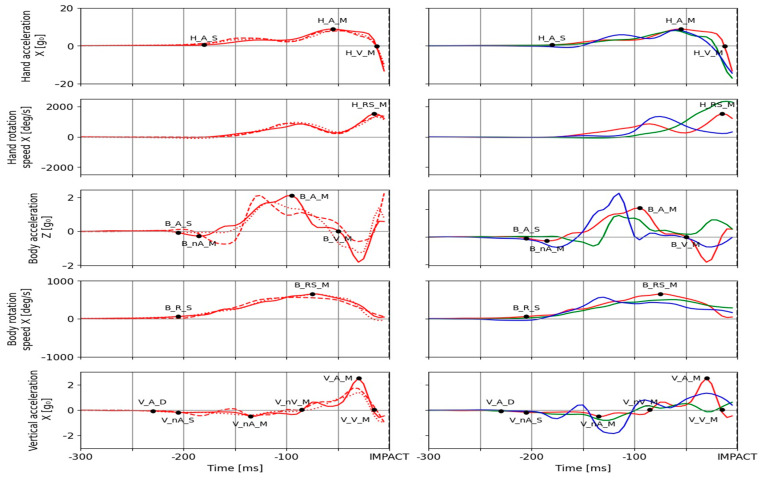
The relevant sensor signals with examined temporal events for the same participant (**left**) and different participants (**right**).

Figure 3 shows a sample of 3 individual strikes performed by the same/different participant with plotted aforementioned temporal events on the corresponding sensor signals. The onset of the hand movement (*H_A_S*) is detected as the threshold value of 0.5 g_0_ on the signal of hand acceleration in the dominant movement axis. The same signal was used for identification of the time of maximal hand acceleration (*H_A_M*) using the peak detection method, while the time for maximal hand velocity (*H_V_M)* was determined a priori as the point of acceleration crossing the zero value. The maximal rotation speed of the hand (*H_RS_M*) was determined from the gyroscope signal for the same axis and was detected as the peak value. This variable was used in order to include the influence of the rotation of the forearm on elbow extension as a possible constraint for maximal performance manifestation. The frontal body acceleration was used for the identification of 4 relevant events, namely (*B_A_S*, *B_nA_M*, *B_A_M*, and *B_V_M*). These events are used for identification of center of gravity (COG) movement onset, time of maximal backward movement as a possible indicator of implementation of the reactive component to the strike execution, and time of maximal frontal body acceleration, respectively. The threshold value for *B_A_S* was −0.2 g_0_ and *B_V_M* was detected a priori. The other two events were detected as peak values. The rotational movement of the pelvis was examined using the start of body rotation (*B_R_S*), detected as the threshold value of 50 deg/s, and maximal body rotation speed (*B_RS_M*) detected as the peak value in the signal of rotational speed. The vertical acceleration of the body is essential for overall movement kinematics as well as for possible early detection of movement as it reflects the changes in the distribution of weight and body support. Thus, the acquired signal of vertical acceleration was used to identify the time of the slightest disturbance (*V_A_D*). The absolute value of the vertical acceleration of 0.05 g_0_ was used as the threshold for detection. Vertical acceleration start (*V_nA_S*) was detected when the threshold value of −0.15 g_0_ was reached. Maximal negative vertical acceleration (*V_nA_M*) was detected as the peak negative value prior to the maximal negative vertical velocity of the body (*V_nV_M)* for which the time of occurrence was known a priori as the acceleration signal crosses the 0. Maximal vertical acceleration (*V_A_M*) was determined as the peak value and maximal vertical velocity (*V_V_M*) was detected a priori from zero crossing. The maximal hand velocity (*MaxHandVel*) is derived from the dominant hand acceleration component by numeric integration. The used sensor signals acquired from 3 trials performed by the same participant in a single testing session are shown in Figure 3—left. The used sensor signals acquired from 3 different participants (single testing session) are shown in Figure 3—right. The examined temporal events are marked on an exemplar strike both Figure 3 left and right.

### 2.5. Statistical Analysis

In the first step of the analysis, the measures of central tendency and data dispersion were determined for the maximal achieved velocity of the hand. The normality of the distribution of the results was determined using the Shapiro–Wilk goodness of fit test. Subsequently, all strikes were categorized into 3 groups in relation to the achieved absolute value of the maximal hand velocity. The results were scaled to a three-point ordinal scale and converted to nominal values used for further analysis. In order to provide 3 groups similar in size for comparison the cut-off value for group division was set to z = ±0.5. The classification methodology was previously described in [37,38,39]. 

In the next step of the analysis, all temporal events were transformed into ranks, thus providing a relative measure of the temporal structure of each strike not affected by the inherent differences in the absolute duration of the movement. The median rank of events was provided. In the final step of the analysis, a step-down approach was adopted. General differences in the temporal structure of the punch were determined using a non-parametric Kruskal–Wallis test, for which a *p* ≤ 0.05 was considered statistically significant. The Mann–Whitney U test was used for pairwise comparisons, i.e., in order to determine the differences between individual groups. In order to provide a more stringent criteria, a *p* ≤ 0.01 was considered statistically significant for post-hoc tests.

All statistical analyses and data processing were performed using Python3 Pandas and SciPy libraries [40,41]. 

## 3. Results and Discussion

The results of descriptive statistical analysis are shown in Table 2. The table includes the results for the overall sample (ALL). The calculated statistics include the mean value (Mean), standard error of the mean (SEM), 95% confidence interval (CI), coefficient of variation (cV), minimum and maximum (Min and Max), as well as the Shapiro–Wilk test statistic and significance (W and Sig.).

The results of the descriptive statistical analysis have shown that the maximal hand velocity (*MaxHandVel*) in the overall sample of the examined strikes was in the range 3.48–9.35 m/s, with the mean value of 6.64 ± 1.02 m/s. The results are normally distributed (*p* = 0.052, W = 0.984) as shown in Table 2. *MaxHandVel* was used as a basis for the division of the overall sample in the following groups: slow—SLW, average—AVG, and fast—FST, using the appropriate classification method [33,34]. The median maximal velocity of the hand achieved by SLW, AVG, and FST group was 5.72, 6.37, and 7.11 m/s, respectively. These results are in line with the previous research by [42,43], which determined maximal wrist velocity of 7.65 ± 0.86 m/s in Malaysian karate athletes, and 8.21 ± 1.6 m/s in expert karate practitioners, respectively, as well as to the related data on punch velocity presented in [44].

In order to provide a relative measure of the temporal structure of the reverse punch not affected by the inherent differences in the absolute duration of the movement, all relevant events in the timeline of the strike were transformed into ranks. The mean rank of all examined events in relation to the *MaxHandVel* group is presented in Table 3 which shows the median rank of the examined temporal events in relation to the group membership. The temporal structure of the strikes categorized in the FST group can be considered preferable and presents an initial model of the optimal temporal structure of the strike.

The results of the Kruskal–Wallis test for general differences of detected events between groups in relation to the maximal velocity of the hand are presented in Table 4. 

The results of the Kruskal–Wallis test have shown statistically significant general differences in the mean rank of *H_A_S* (x^2^ = 10.31, *p* = 0.006), *H_V_M* (x^2^ = 8.64, *p* = 0.013), *B_V_M* (x^2^ = 7.66, *p* = 0.022), *H_A_M* (x^2^ = 10.37, *p* = 0.006), *B_A_M* (x^2^ =7.25, *p* = 0.027), and *V_A_D* (x^2^ = 0.45, *p* = 0.009) between the three examined groups in relation to the achieved maximal hand velocity (Table 4). This clearly indicates that there are differences in motion sequencing, i.e., the temporal structure of the strike, between the three examined groups.

Table 5 presents the results of the Mann–Whitney U test for pairwise differences in the mean rank of individual events between the individual group pairs in relation to the maximal velocity of the hand.

The results of the Mann–Whitney test have shown that the difference in *H_A_S* is statistically significant (U = 1012, *p* = 0.001) between the groups SLW (Mdn = 4.5) and FST (Mdn = 2). The calculated mean rank of 66.91 and 47.15 for the SLW and FST group, respectively, indicates that the FST group initiates the hand movement at an earlier point in the overall movement timeline. A statistically significant difference in *H_V_M* was determined (U = 1079.50, *p* = 0.004) for the SLW (Mdn = 14.5) and FST (Mdn = 15) groups. Based on the calculated mean rank values of 47.37 and 64.70 for the respective groups, it can be argued that the FST group achieves the maximal velocity of the hand at a time closer to the impact. The determined difference in *B_V_M* rank was statistically significant (U = 1136.00, *p* = 0.010) for SLW and FST groups (Mdn = 12), for both groups. The calculated mean rank value for SLW group was 64.57, while for the FST group the mean rank value was 49.25. This indicates that the SLW group reaches the maximal velocity of the body at a later point in the movement timeline when compared to the FST group. For the *H_A_M,* the determined difference in event rank was statistically significant (U = 1029.50, *p* = 0.002) between groups SLW (Mdn = 10) and FST (Mdn = 11). The mean rank for the SLW group was 46.42 while for the FST group it was 65.55, indicating that the FST group achieves maximal acceleration of the hand later than the SLW group.

Statistically significant differences between AVG and FST groups were determined for *B_A_M* (U = 1099.50, *p* = 0.005; both Mdn = 8) and *V_A_D* (U = 1238.00, *p* = 0.006; both Mdn = 1) events. The mean rank value for *B_A_M* was 65.25 for the AVG group and 48.64 for the FST group, while the mean rank value for *V_A_D* was 62.64 and 50.98, respectively. This indicates that the *B_A_M* and *V_A_D* are achieved at a later time in the movement timeline for the AVG group.

The presented results support the initial hypothesis regarding the differences related to the movement temporal structure, i.e., the timeline of the relevant events, between the strikes of different velocities. This is most likely due to the differences in synchronization of the sequential sub-movements which certainly affects the movement kinematics [9]. The present paper has determined that it is possible to detect these differences using the presented methodology. In relation to the previous research, it is necessary to point out the fact that an experimental setup with only two IMUs provided information regarding the temporal characteristics of three key components that contribute to the punch force and velocity, namely drive off the ground by the legs, rotation of the trunk, and action of the arm muscles [9,10,41,45,46]. These are represented by the events acquired from the vertical and frontal acceleration of the body; the rotational speed of the body; and acceleration and rotational speed of the hand, respectively. Further research on the temporal structure of the reverse punch, as well as some other related movements, is recommended.

## 4. Conclusions

This paper has proposed a new method for measurement of the synchronization of the movement kinematics, using two IMUs mounted on the body of the athlete. The established differences indicate that the strikes that achieved a high maximal velocity of the hand have a different temporal pattern of relevant events when compared to the average and low-velocity strikes. The presented results point out the effects of the possible differences in the control mechanisms governing the strike. In addition, these results indicate that the presented methodology is suitable for monitoring the structure of the movement during repetitive execution in live practice, which affects the acquisition and stabilization of the preferred movement patterns. In this sense, the measurement of movement temporal structure using IMU provides new, more in-depth, and thus more relevant insights into factors affecting performance. This paper does not take into account the differences related to the movement of the knee, shoulder, and elbow joints, which contribute to the overall kinematics of the reverse punch. Further research on the temporal structure of the reverse punch and related movements using a larger number of sensor units may provide additional information. As the contribution of the preceding segments is aggregated toward the endpoint of the kinetic chain, we consider a two-point setup covering the movement of the body COG and the hand to be an optimal solution. This allows for sufficient level of decomposition of the movement in relation to main factors that contribute to it and is supported by the results of our study. 

## Figures and Tables

**Table 1:** Detailed description of the events.

Abbreviation	Description	Sensor	Signal	Axis	Detection Method
*V_A_D*	Overall movement start; First vertical disturbance	BODY	lin. acceleration	X	threshold
*H_A_S*	Hand movement start	HAND	acceleration	X	threshold
*V_nA_S*	Vertical displacement start; The start of the underweight phase of the movement	BODY	lin. acceleration	X	threshold
*B_R_S*	Hip rotation start	BODY	rotation speed	X	threshold
*B_A_S*	Frontal acceleration start	BODY	lin. acceleration	Z	threshold
*B_nA_M*	Maximal backward body acceleration	BODY	lin. acceleration	Z	peak
*V_nA_M*	Maximal vertical acceleration of the body in the underweight phase	BODY	lin. acceleration	X	peak
*B_A_M*	Maximal forward acceleration of the body	BODY	lin. acceleration	Z	peak
*V_nV_M*	Maximal negative vertical velocity; Start of countermovement stretching phase	BODY	lin. acceleration	X	zero crossing
*B_RS_M*	Maximal hip rotation speed	BODY	rotation speed	X	peak
*H_A_M*	Maximal forward hand acceleration	HAND	acceleration	X	peak
*B_V_M*	Maximal forward body acceleration	BODY	lin. acceleration	Z	zero crossing
*H_RS_M*	Maximal rotation speed of the forearm	HAND	rotation speed	X	peak
*V_A_M*	Maximal vertical acceleration; Start of propulsion	BODY	lin. acceleration	X	peak
*H_V_M*	Maximal hand velocity	HAND	acceleration	X	zero crossing
*V_V_M*	Maximal vertical velocity; End of vertical propulsion	BODY	lin. acceleration	X	zero crossing
MaxHandVel	Maximal velocity of the hand	HAND	acceleration	X	num. integration

**Table 2:** The descriptive statistics for the maximal achieved hand velocity in relation to the overall sample.

Statistics											
	Group	N	Mean	SEM	95% CI	SD	cV	Min	Max	W	Sig.
*MaxHandVel* [m/s]	ALL	165	6.44	0.08	6.28–6.60	1.02	15.87	3.48	9.35	0.984	0.052

**Table 3:** The median rank of all measured events in the reverse punch timeline in relation to the examined sub-samples.

Event Median Rank								
Group	*V_A_D*	*H_A_S*	*V_nA_S*	*B_R_S*	*B_A_S*	*B_nA_M*	*V_nA_M*	*B_A_M*
SLW	1	4.5	3	4.5	2	6	7	8
AVG	1	3.5	3.5	3.5	3.5	6	7	8.5
FST	1	2	3.5	3.5	5	6	7	8
	*V_nV_M*	*B_RS_M*	*H_A_M*	*B_V_M*	*H_RS_M*	*V_A_M*	*H_V_M*	*V_V_M*
SLW	9	11	10	12	13	14.5	14.5	16
AVG	8.5	10	11	13	12	14.5	14.5	16
FST	9	10	11	12	13	14	15	16

**Table 4:** The general differences in the temporal structure of the reverse punch between the strikes classified as fast, average, and slow in relation to the achieved maximal hand velocity.

Kruskal–Wallis Test								
	*H_A_S*	*B_R_S*	*B_RS_M*	*H_RS_M*	*H_V_M*	*B_V_M*	*H_A_M*	*B_A_S*
Chi-Square	10.31	0.74	0.16	1.36	8.64	7.66	10.37	4.12
df	2.00	2.00	2.00	2.00	2.00	2.00	2.00	2.00
Sig.	0.006	0.690	0.925	0.507	0.013	0.022	0.006	0.127
	*B_nA_M*	*B_A_M*	*V_A_D*	*V_nA_S*	*V_nA_M*	*V_nV_M*	*V_A_M*	*V_V_M*
Chi-Square	2.34	7.25	9.45	0.89	5.16	3.00	0.29	1.12
df	2.00	2.00	2.00	2.00	2.00	2.00	2.00	2.00
Sig.	0.310	0.027	0.009	0.641	0.076	0.223	0.866	0.571

**Table 5:** The pairwise comparisons of the temporal structure of the reverse punch between the strikes classified as fast, average, and slow in relation to the achieved maximal hand velocity.

Mann–Whitney									
		*H_A_S*	*B_R_S*	*B_RS_M*	*H_RS_M*	*H_V_M*	*B_V_M*	*H_A_M*	*B_A_S*
SLW-AVG	U	1232.00	1349.50	1379.50	1279.00	1275.00	1376.50	1149.00	1183.00
	Sig.	0.272	0.726	0.872	0.424	0.398	0.856	0.100	0.158
SLW-FST	U	1012.00	1410.50	1528.00	1370.50	1079.50	1136.00	1029.50	1216.00
	Sig.	0.001	0.369	0.831	0.257	0.004	0.010	0.002	0.041
AVG-FST	U	1243.50	1493.00	1496.50	1508.00	1245.00	1198.50	1291.50	1524.50
	Sig.	0.060	0.679	0.688	0.745	0.057	0.030	0.105	0.819
		*B_nA_M*	*B_A_M*	*V_A_D*	*V_nA_S*	*V_nA_M*	*V_nV_M*	*V_A_M*	*V_V_M*
SLW-AVG	U	1359.50	1269.50	1152.00	1396.00	1079.50	1147.50	1336.50	1343.00
	Sig.	0.775	0.377	0.027	0.957	0.031	0.088	0.659	0.569
SLW-FST	U	1314.00	1319.00	1512.50	1404.50	1520.00	1391.50	1481.50	1432.50
	Sig.	0.142	0.143	0.593	0.351	0.789	0.290	0.626	0.287
AVG-FST	U	1375.50	1099.50	1238.00	1449.50	1274.00	1446.00	1555.00	1505.50
	Sig.	0.270	0.005	0.006	0.504	0.080	0.470	0.960	0.647

## Data Availability

The data presented in this study are available on request from the corresponding author. The data are not publicly available due to privacy restrictions.
